# Human electrocortical dynamics while stepping over obstacles

**DOI:** 10.1038/s41598-019-41131-2

**Published:** 2019-03-18

**Authors:** Andrew D. Nordin, W. David Hairston, Daniel P. Ferris

**Affiliations:** 1 J. Crayton Pruitt Family Department of Biomedical Engineering, University of Florida, Gainesville, USA; 2 Human Research and Engineering Directorate, U.S. Army Research Laboratory, Aberdeen Proving Ground, USA

## Abstract

To better understand human brain dynamics during visually guided locomotion, we developed a method of removing motion artifacts from mobile electroencephalography (EEG) and studied human subjects walking and running over obstacles on a treadmill. We constructed a novel dual-layer EEG electrode system to isolate electrocortical signals, and then validated the system using an electrical head phantom and robotic motion platform. We collected data from young healthy subjects walking and running on a treadmill while they encountered unexpected obstacles to step over. Supplementary motor area and premotor cortex had spectral power increases within ~200 ms after object appearance in delta, theta, and alpha frequency bands (3–13 Hz). That activity was followed by similar posterior parietal cortex spectral power increase that decreased in lag time with increasing locomotion speed. The sequence of activation suggests that supplementary motor area and premotor cortex interrupted the gait cycle, while posterior parietal cortex tracked obstacle location for planning foot placement nearly two steps ahead of reaching the obstacle. Together, these results highlight advantages of adopting dual-layer mobile EEG, which should greatly facilitate the study of human brain dynamics in physically active real-world settings and tasks.

## Introduction

Encountering unpredictable terrain is a daily occurrence, but we understand relatively little about how the human brain responds to and overcomes obstacles during locomotion. Studies on cats have revealed information about how quadrupeds process visual information for coordinating limb motions when stepping over obstacles^1–3^. The motor cortex is involved in executing these locomotor adjustments^2–4^ but many cortical structures assist in planning gait modifications^1^. Posterior parietal cortex, in particular, has neuronal firing that appears to track the distance to approaching obstacles during visually guided locomotion^5^. Within the human brain, direct evidence of dynamic brain responses to obstacles during locomotion has been difficult to obtain due to neuroimaging limitations, but a recent review of brain dynamics during human obstacle navigation^6^ has suggested there may be contrasting neural pathways for slow versus fast locomotor adjustments, involving both cortical and subcortical structures.

Slow voluntary motor adjustments have been attributed to prefrontal, premotor, and primary motor cortices, while fast automatic adjustments have been linked to posterior parietal cortex^7–9^ and subcortical pathways involving superior colliculi, pontine nucleus, or cerebellum^10–16^. Much of the evidence for these conclusions is indirect, as virtually all current brain imaging modalities require subjects to remain stationary to avoid severe artifacts. To circumvent these limitations, upper limb tasks and imagined movements have traditionally been studied using large, costly brain imaging modalities such as functional magnetic resonance imaging^17–20^ or positron emission tomography^21,22^ that measure blood flow-related changes in the brain over several seconds, or magnetoencephalography that measures magnetic induction due to neuronal activity with millisecond precision^23–25^. To study neural control of real locomotion and obstacle avoidance, researchers have therefore relied on lower limb dynamics and electrical muscle activation timing^6,26–28^, and portable brain imaging methods that measure hemodynamic responses with low time resolution, such as functional near infrared spectroscopy^22,29–31^.

Electroencephalography (EEG) provides a fast-timescale, non-invasive measure of human brain dynamics that has been used to understand and decode cortical activity during controlled upper limb movements^32–34^. Fortunately, EEG hardware can be worn portably during locomotion, but mobile EEG signals are prone to motion artifact contamination at fast walking and running speeds^35–38^. Many studies have now shown electrocortical spectral power fluctuations tied to the gait cycle in brain structures including occipital lobe, supplementary motor area, anterior cingulate, posterior parietal, prefrontal, premotor, and sensorimotor cortices^39–49^. However, challenges in separating motion artifacts from these brain signals has led to uncertainty in their interpretation^36,39,50,51^. Data processing pipelines that use independent component analysis and source localization can separate motion artifacts from electrical brain activity for some walking speeds, but are not successful for fast walking or running^51^.

Within these constraints, EEG recordings during controlled gait perturbations have shown spectral power changes in frontal, central midline, and parietal brain regions following externally cued shifts in step rate and length^52^. These results aligned with subdural recordings from electrocorticography^53^ and scalp EEG^54^ during upper limb movements, showing that electrocortical responses occur after an external cue, but before movement interruption. Motor adjustments therefore exhibit top down inhibitory control, which can be measured from EEG signals during gait. Mobile EEG studies have yet to uncover motor cortical signals during human obstacle navigation^55^, mostly due to concerns with gait related motion artifacts.

To overcome limitations associated with motion artifacts in mobile EEG recordings, it may be possible to combine multiple EEG electrodes in a dual-layer setup where one layer records a mix of biological content and motion artifacts, and the second layer records pure motion artifacts^56^. Dual-layer EEG recordings have been used in simultaneous EEG-fMRI to remove gradient and motion artifacts from EEG data when exposed to electromagnetic interference from the MRI scanner^57,58^ and the feasibility of this approach was recently demonstrated for mobile EEG hardware^56^. By simultaneously recording normal scalp EEG along with isolated noise recordings from matched pairs of electrically isolated electrodes, Nordin and colleagues effectively cleaned artifact contaminated mobile EEG data^56^.

The purpose of this study was to determine if we could record and isolate human electrocortical activity when encountering unexpected obstacles across a range of locomotion speeds. To overcome limitations associated with motion artifacts contaminating EEG signals, we devised hardware and signal processing solutions for motion artifact removal and validated this approach prior to human trials. We used novel dual-layer electrodes that consisted of matched pairs of EEG and noise electrodes. The two electrodes were mechanically connected, but electrically isolated so that the secondary sensors exclusively captured motion artifacts and electrical noise.

## Materials and Methods

### Dual-layer EEG Validation

**Figure 1:**
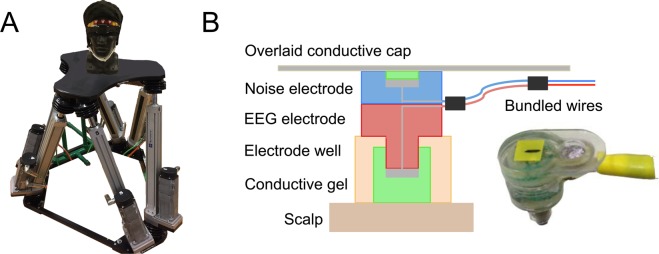
(**A**) Phantom head with artificial electrical brain signals, mounted to a six degree of freedom motion platform that reproduced human head trajectories while walking. (**B**) Dual electrode configuration with matched EEG and noise electrode pair.

To test our hardware and signal processing approaches, we built a human head shaped phantom device using dental plaster^56,59,60^. The phantom contained six spatially distributed electrical dipolar antennas. A connected USB digital to analog converter (USB-3101FS, Measurement Computing, Norton, MA) was used to generate artificial brain signals in LabVIEW (100 Hz sample rate, National Instruments, Austin, TX). Artificial brain signals consisted of randomly occurring and overlapping 500 ms sinusoidal pulses with differing frequency content at each antenna (7, 13, 17, 23, 29, 37 Hz). To create realistic motion, we reproduced human head movements using a Notus hexapod (Fig. 1A) (Symétrie, Nimes, France) based on data collected using a forehead mounted inertial measurement unit (IMU, 128 Hz sample rate, APDM, Portland, OR) from a subject walking for 10-minutes at 0.4, 0.8, 1.0, 1.6, 2.0 m/s on a split belt treadmill (Bertec, Columbus, OH).

To create a set of matched dual electrode pairs, we assembled an array of EEG electrodes using BioSemi ActiveTwo hardware (Amsterdam, The Netherlands), sampling at 512 Hz. Each scalp interfacing pin type electrode was rigidly coupled to an inverted flat type electrode, which exclusively captured motion artifacts and electrical noise, without electrical connection to the scalp (Fig. 1B). We assembled a 40-channel dual electrode array by securing wires from each electrode pair and bundling wires among channels. Scalp and inverted electrodes were recorded from separate daisy-chained BioSemi systems. During testing, we placed a correctly sized EEG cap on the head and inserted conductive gel into each cap well, followed by each scalp electrode. Because of the electrical independence of the inverted noise electrodes, we fit a custom conductive fabric cap (Eeonyx, Pinole, CA) over all electrodes and inserted conductive gel between the fabric and noise electrode using a syringe, forming an external artificial skin circuit. For both the EEG and noise sensors, we confirmed electrodes offsets were below 20 mV. To compare the ability of dual-layer EEG to remove motion artifacts relative to a standard single-layer EEG setup, we repeated the experimental procedure using 40 BioSemi ActiveTwo electrodes at the same relative channel locations.

Data were processed in MATLAB (MathWorks, Natick, MA) using scripts based on EEGLab 14.1.1b functions (http://sccn.ucsd.edu/eeglab)^61^. For both single and dual-layer recordings, we high pass filtered the data at 1 Hz, concatenated speed conditions, and re-referenced the scalp channel data to common average. When processing dual-layer EEG, noise channels were separately referenced to common average before merging scalp and noise channels into a single 80-channel matrix. Next, we performed Infomax independent component analysis separately on the single (40-channel) and dual-layer (80-channel) EEG datasets to recover spatially fixed, maximally temporally independent sources from the channel data^62^. We compared extracted independent components to the ground truth input signals using cross correlation and examined the power spectra of each component to assess our ability to recover independent source signals from single and dual-layer EEG during motion. Because each artificial brain antenna broadcast distinct frequency content, the recovered independent components should show a spectral profile with a single source frequency peak and negligible motion artifacts.

### Human Obstacle Navigation Experiment

Prior to human testing, we modified our dual electrode array to include 128-EEG and 40-noise electrodes, as well as 8 electromyography neck electrodes (176 total channels, 88-single electrode EEG and 40-dual electrode EEG channels), and bundled all wires into a single rear-exiting cable using Velcro straps. The experimental protocol was approved by the Institutional Review Boards at University of Michigan and University of Florida, and conformed to the Declaration of Helsinki and national guidelines. Nine (6 male, 3 female) healthy, right limb dominant, subjects participated after providing informed consent, including the use of identifying images for publication. Subject preparation followed previous protocols from our lab^39^, including electrode location digitization (Zebris, Isny, Germany). During treadmill testing, we placed the EEG systems on a bodyweight support system above the subject’s head^39^. We collected biomechanical data from an inertial measurement unit on the subject’s forehead (128 Hz sample rate, APDM, Inc., Portland, OR), 10-camera system capturing lower extremity kinematics (100 Hz sample rate, Vicon, UK), and a force-instrumented treadmill (1000 Hz sample rate, Bertec, Columbus, OH). The data were synchronized using an analog pulse generated by the inertial measurement unit and timing gates.

Subjects completed six speed conditions, lasting 3 minutes each, including walking at 0.5, 1.0, 1.5, and 2.0 m/s, and running at 2.0 and 2.5 m/s. Condition order was randomized with rest periods between. We asked subjects to walk naturally, but to avoid unnecessary head motions, jaw clenching, or eye blinking. Subjects were also instructed to step over foam obstacles that would randomly appear from behind a white curtain at the front of the treadmill, spanning the belt width (9 obstacles per condition). Obstacles were tracked with motion capture and their appearance on the treadmill was synchronized to motion capture via a timing gate that sent an analog signal to both the motion capture data acquisition board (1000 Hz sample rate, Vicon Giganet, UK) and EEG recording.

### Human Obstacle Navigation Analysis

To account for magnitude differences between EEG and noise recordings, we scaled the amplitude of each noise channel to its EEG pair using Fast Fourier Transform in a 500 ms sliding window with 94% overlap^56^. We scaled the magnitude of the noise Fourier coefficients to match those in the EEG signal using the median values within each window and reconstructed an amplitude matched noise signal. This step accounted for resistivity differences between human scalp and the conductive fabric. All data were high pass filtered at 1 Hz and channels with large artifacts were rejected based on statistical criteria (range, standard deviation, kurtosis)^39^. We retained an average of 117 ± 5 EEG channels, 38 ± 2 noise channels, and 7–8 electromyography channels. Each dataset was then separately re-referenced to common average, merged by stacking channels, and down sampled to 256 Hz prior to applying an adaptive mixture independent component analysis^63^. Separately average referencing each dataset avoided introducing pure noise and electrical muscle activity into our EEG data through a single common average, which showed poor source separation during pilot testing.

We rejected artifact contaminated independent components with flat power spectra (linear slope ≥ −0.06) or spectra matching noise or electromyography sensors (polynomial fit R^2^ ≥ 0.99). To select and test these rejection criteria, we performed an adaptive mixture independent component analysis exclusively on noise and electromyography data and confirmed no independent components were retained. After applying these rejection criteria to an adaptive mixture independent component analysis performed on the complete electrode set, we retained an average of 59 ± 17 independent components per subject.

Next, we modeled ICs as equivalent current dipoles using a three layer boundary element model and subject-specific anatomical magnetic resonance image warped to the Montreal Neurological Institute standard brain (Montreal, Canada) using DIPFIT and Fieldtrip functions in EEGLab^64^. Subsequently, independent components with equivalent current dipoles explaining greater than 85% of the scalp map were retained, leaving an average of 10 ± 9 components per subject. For group analysis, we used *k*-means clustering (*k = *7, outlier components > 3 standard deviations from cluster centroid were excluded)^47^ on vectors jointly coding dipole locations, scalp maps, and power spectra similarities in EEGLab^65,66^. Cortical clusters with independent components from more than 50% of the subjects were further analyzed^39^. To avoid artificially inflating sample size by including multiple components per subject in a cluster, we retained a single component per subject in each cluster based on dipole location, scalp map and explained variance (2–3 components per cluster were excluded).

**Figure 2:**
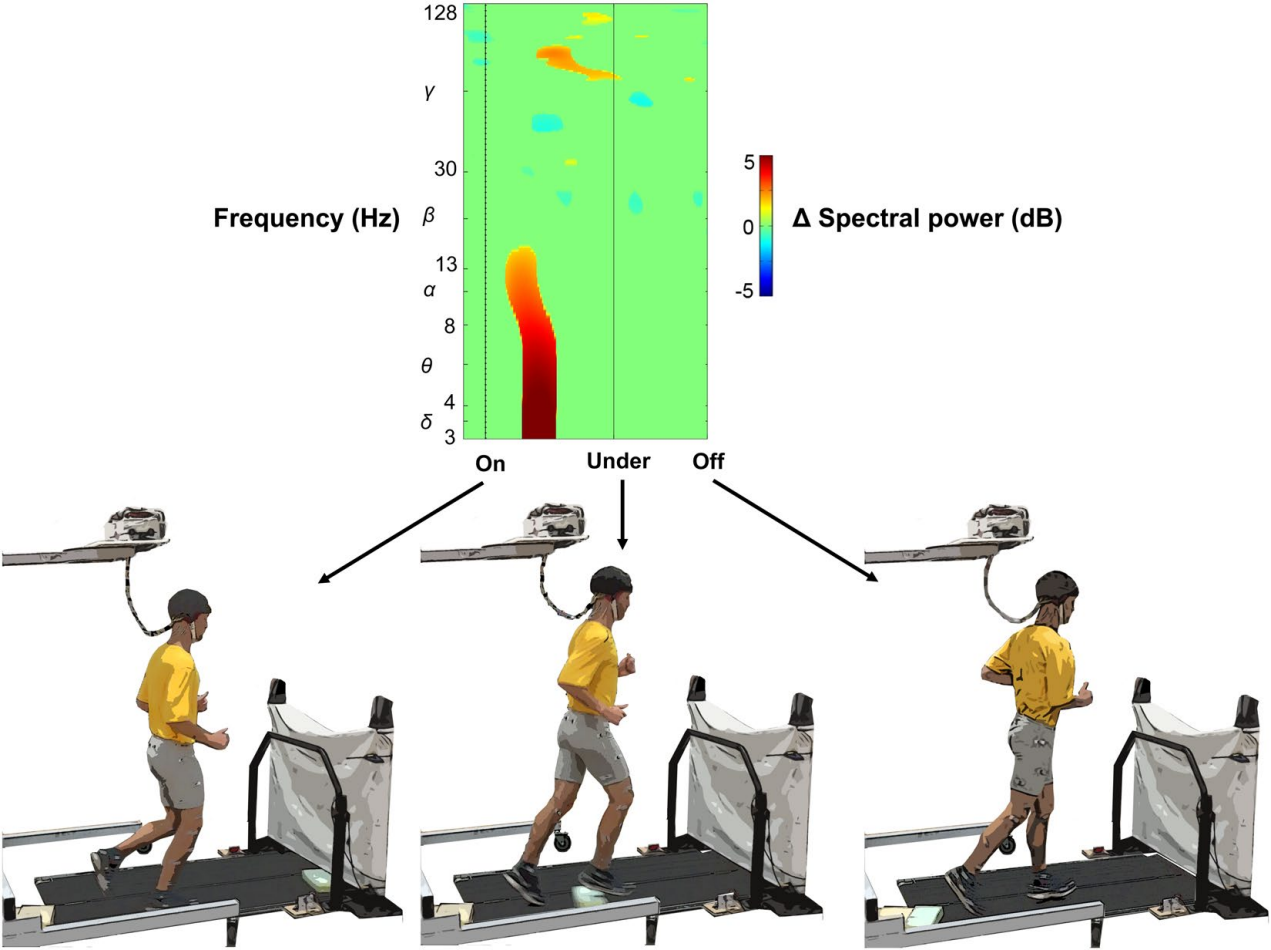
Obstacle events used to time warp event related spectral perturbation plots. Obstacle events: obstacle appears on the treadmill belt (On), obstacle directly under the subject (Under), and obstacle leaves the treadmill (Off).

We then performed time-frequency analysis on our independent components by extracting obstacle events from the timing gate sync signals and motion capture data, which were low pass filtered (6 Hz cutoff, 4^th^ order Butterworth), temporally aligned, and resampled to match EEG data. The three obstacle events included: 1) when the obstacle appeared on the treadmill belt (On), 2) when the obstacle was under the subject (Under, anterior-posterior hip-obstacle intersection), and 3) when the obstacle left the treadmill belt (Off) (Fig. 2). Single-trial spectrograms were computed for each independent component and the mean log spectrum was subtracted from all time points in each condition, creating spectral power changes relative to baseline, or event related spectral perturbations^67^. We averaged the event related spectral perturbation across independent components in a cluster to create a grand mean event related spectral perturbation for each condition. Each event related spectral perturbation was linearly time warped to the epoch events (On, Under, Off) (Fig. 2).

To compare neural response timings across gait speeds and cortical clusters, we extracted the earliest and most prominent (largest number of connected pixels) change in spectral power (activation onset) following obstacle appearance using significance masked event related spectral perturbations computed using the same methods above for each independent component in a cluster. We also identified the anterior-posterior distance between the subject’s hip marker and the obstacle at the time of activation onset (distance to contact). For both activation onset and distance to contact, Friedman tests were performed with Tukey-Kramer adjustments for multiple comparisons, across gait speeds for each cortical cluster, and across cortical clusters for each gait speed (*α* = 0.05).

The gait phase when neural activation occurred was assessed using vertical ground reaction forces synced to the EEG data. Gait event timing (heel contact and toe off) was extracted for each obstacle event and aggregated among subjects at each locomotion speed (mean ± standard error) to determine when neural activation occurred in the steps before and after overstepping the obstacle.

## Results

### Dual-layer EEG Validation

**Figure 3:**
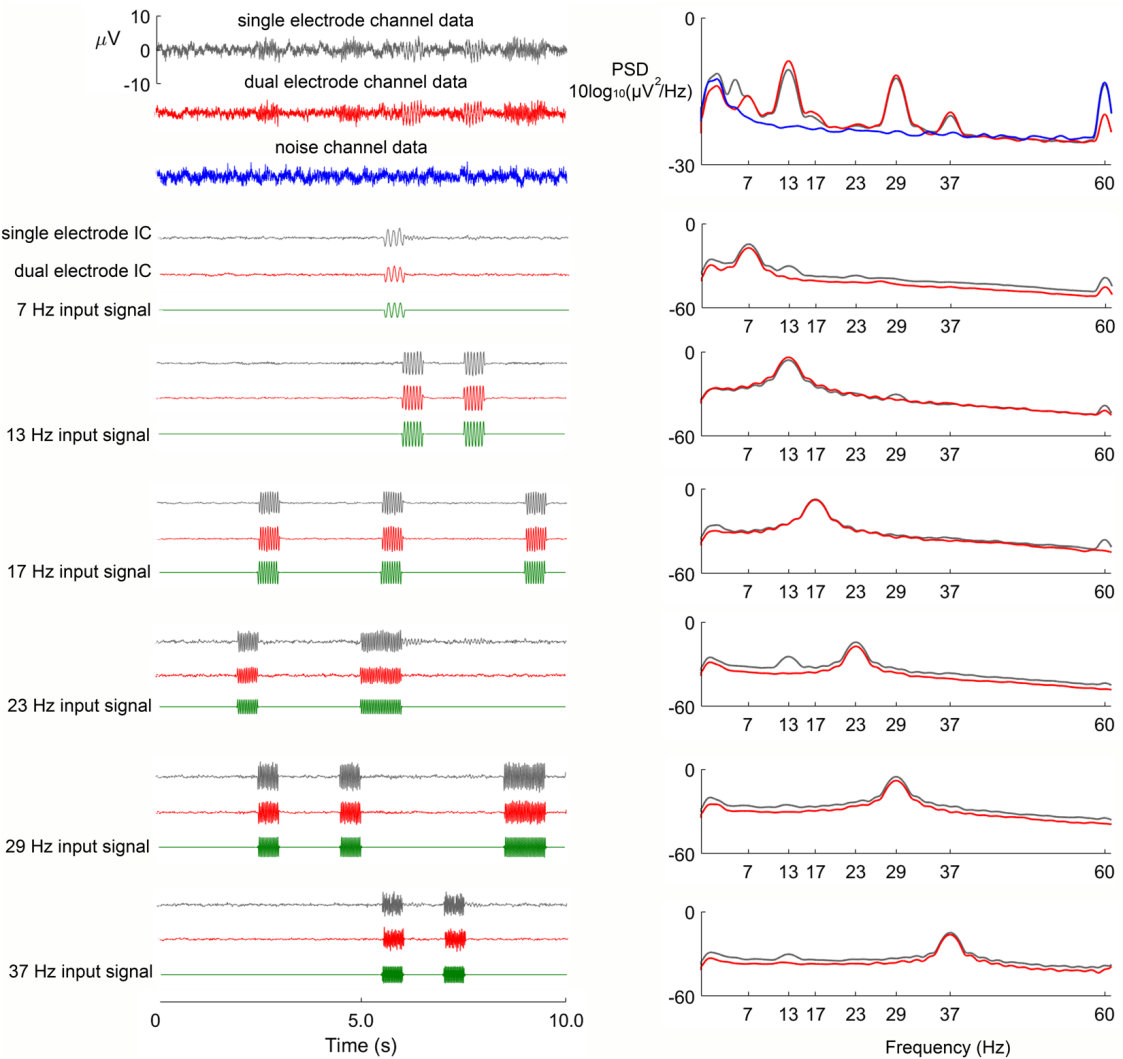
Single and dual-layer EEG comparisons before and after performing independent component analysis (ICA) on artificial brain signals broadcast through the human head phantom during motion. Exemplar time series EEG data (left) and power spectral density (PSD; right) collected during reproduced 2.0 m/s walking.

By merging EEG data from the primary scalp sensors with noise recordings from the secondary sensors into independent component analysis, we recovered artifact-free ground truth input signals from dual-layer EEG. Cross correlation (*r*) between input signals and recovered independent components was 0.92 ± 0.26 for dual-layer EEG and 0.89 ± 0.26 for standard single-layer EEG across gait speeds (stationary through 2.0 m/s). Although cross correlation was relatively high in each case, standard single-layer EEG recordings failed to return independent components with distinct frequency content, while dual layer EEG successfully returned the ground truth input signals (see power spectra in Fig. 3, right column).

### Human Obstacle Navigation

**Figure 4:**
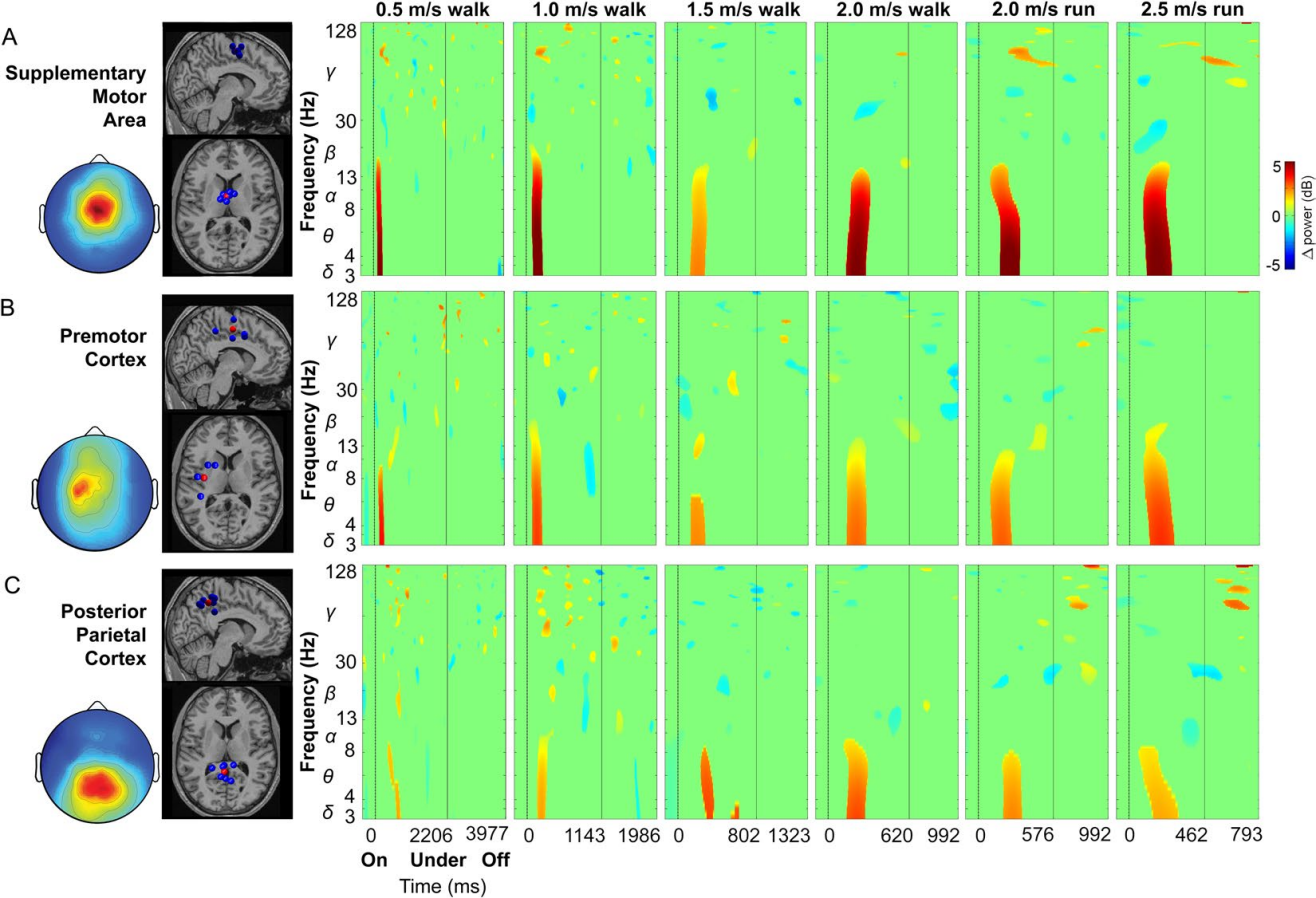
Cortical clusters and event related spectral perturbation plots by speed. (**A**) Supplementary motor area (6 subjects and components), (**B**) Premotor cortex (5 subjects and components), (**C**) Posterior parietal cortex (7 subjects and components). (Left) Cortical cluster scalp map and dipole locations (dipole cluster centroid: red, subject components: blue) and (Right) mean cluster event related spectral perturbation plots at each speed. Significance masked (*p* < 0.05).

We obtained three cortical clusters with independent components from more than 50% of the subjects in supplementary motor area, premotor cortex, and posterior parietal cortex (Fig. 4). Time warped event related spectral perturbation plots for each cortical cluster and gait speed revealed significant increases in spectral power for delta, theta, and alpha bands (3–13 Hz) in supplementary motor area, premotor, and posterior parietal cortex following obstacle appearance, but preceding the step over the obstacle (Fig. 4).

**Figure 5:**
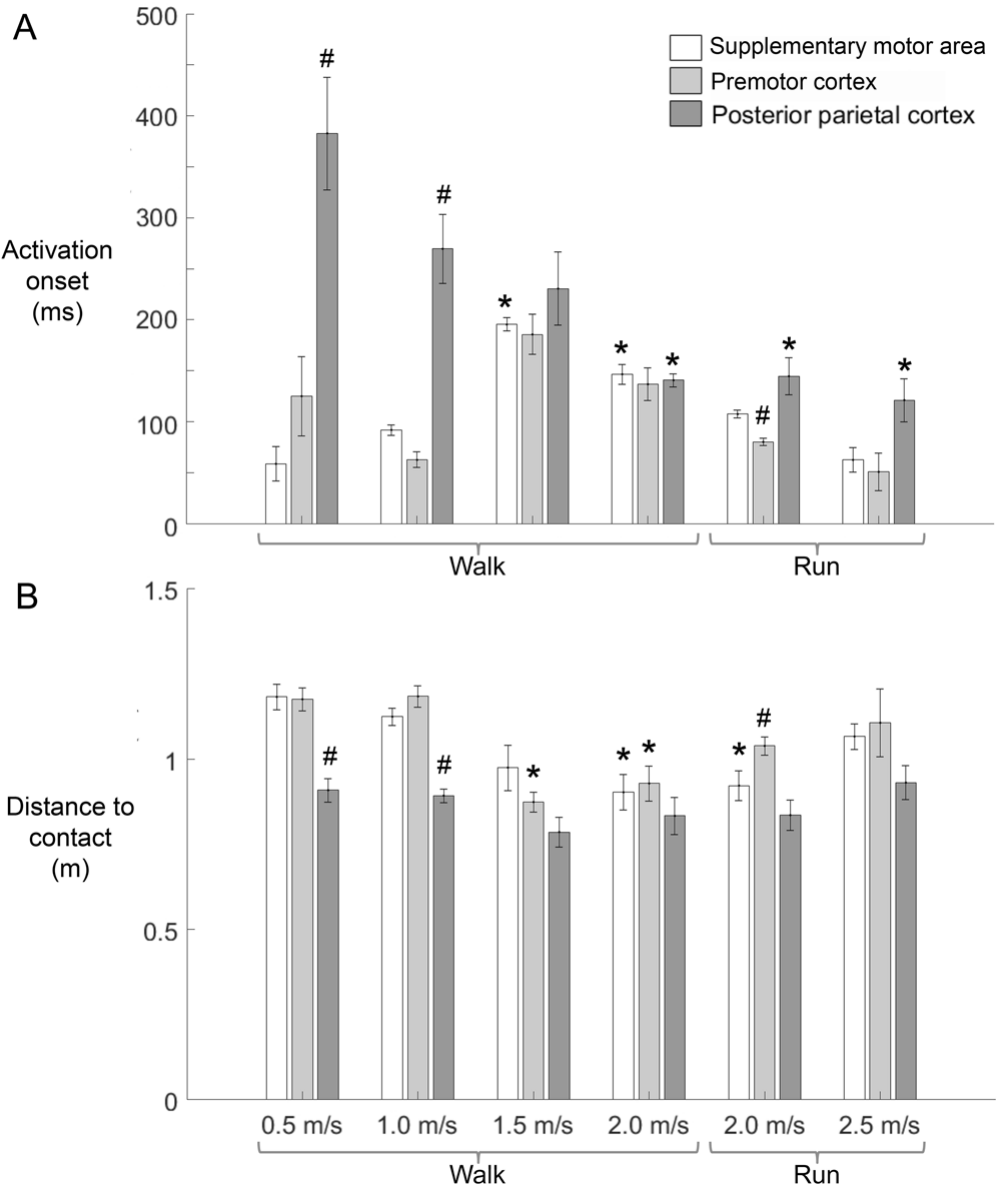
Summaries of activation onset time and distance to contact by speed and cortical cluster. (**A**) Activation onset time: earliest significant spectral power change, and (**B**) distance to contact: distance between the obstacle and subject at activation onset. Mean ± standard error. ^#^cortical clusters differ at given speed, *speeds differ within cortical cluster. Friedman tests by speed and cortical cluster with Tukey-Kramer adjustments for multiple comparisons (*p* < 0.05).

Friedman tests indicated that posterior parietal cortex and supplementary motor area spectral power increase onset was significantly different depending on speed (Fig. 5A). Walking at 2.0 m/s and during running, posterior parietal cortex spectral power increase occurred earlier relative to slower walking conditions (*χ*^2^_(5)_ = 24.3, *p* < 0.001; pairwise comparisons: *p ≤ 0.032). In contrast, supplementary motor area synchronization occurred later during 1.5 and 2.0 m/s walking compared to slower walking and running (*χ*^2^_(5)_ = 24.2, *p* < 0.001; pairwise comparisons: *p ≤ 0.039). Comparisons among cortical clusters showed that posterior parietal cortex spectral power increase timing was later relative to supplementary motor area and premotor cortex during 0.5 and 1.0 m/s walking (*χ*^2^_(2)_ = 12.3, *p* = 0.002; pairwise comparisons: ^#^*p* ≤ 0.015), and 2.0 m/s running (*χ*^2^_(2)_ = 11.1, *p* = 0.0038; pairwise comparisons: ^#^*p* ≤ 0.043).

Posterior parietal cortex spectral power increase occurred at similar distance to contact with the obstacle at each locomotion speed (Fig. 5B). Supplementary motor area and premotor cortex showed small but statistically significant decreases in distance to obstacle contact when there was a spectral power increase during 2.0 m/s walking and running (*χ*^2^_(5)_ = 21.1, *p* < 0.001; pairwise comparisons: *p ≤ 0.040), and 1.5 and 2.0 m/s walking, respectively (*χ*^2^_(5)_ = 18.8, *p* = 0.002; pairwise comparisons: *p ≤ 0.047). Compared to supplementary motor area and premotor cortex, later posterior parietal cortex synchronization occurred when the obstacle was closer to the subject at 0.5 and 1.0 m/s (*χ*^2^_(2)_ = 11.1, *p* = 0.038; pairwise comparisons: ^#^*p* ≤ 0.043).

**Figure 6:**
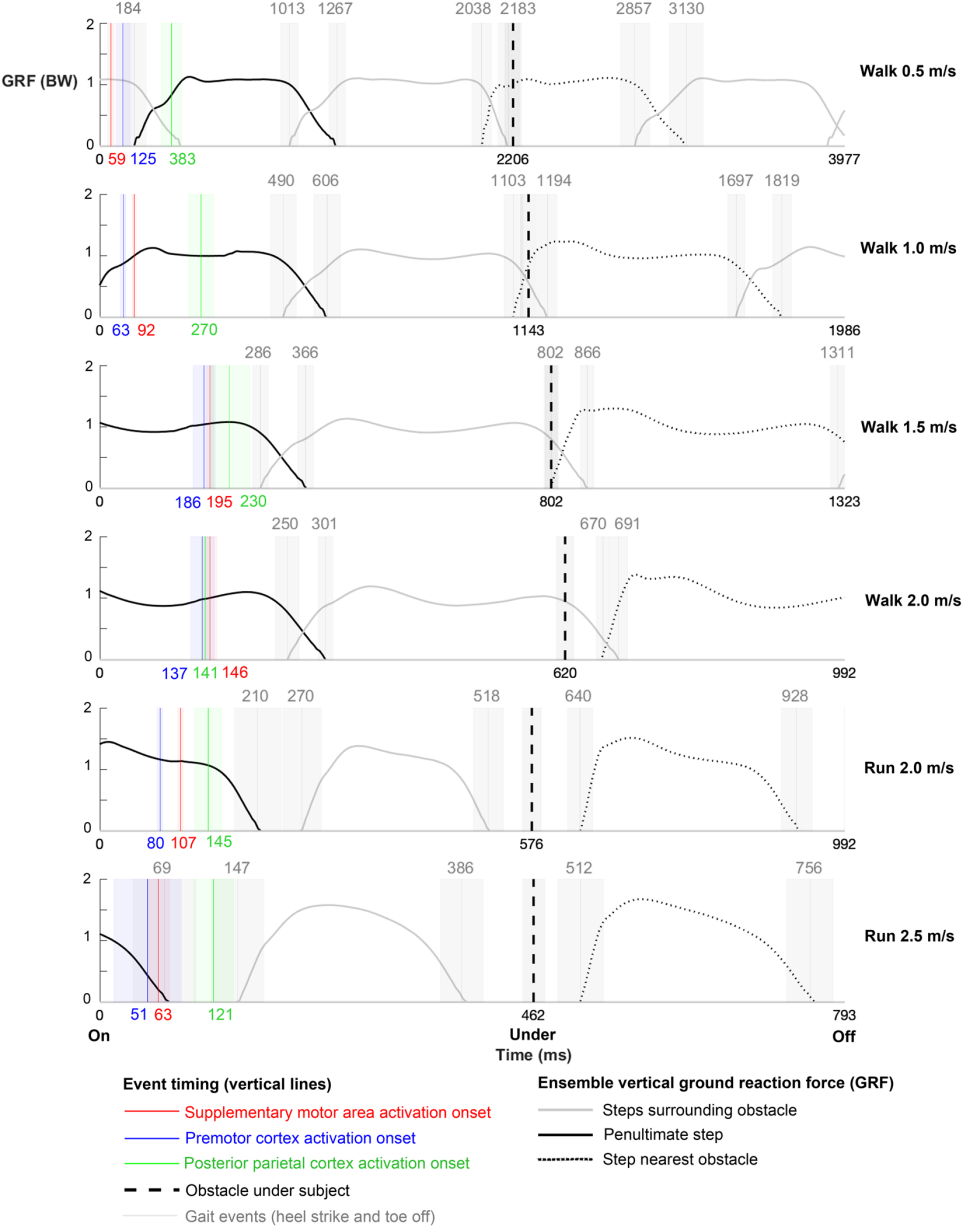
Neural activation timing (mean ± standard error) relative to gait events (heel strike and toe off) when overstepping obstacles at each locomotion speed. Ensemble vertical ground reaction force (GRF, BW: bodyweight) curves aligned and time warped to mean gait event timing (milliseconds).

On average, cortical spectral power increase occurred in the penultimate step before placing the support foot nearest the obstacle (in front or behind the obstacle), except in 2.5 m/s running (Fig. 6). Similar spectral power timing relative to the gait events was the result of longer stance times at slower locomotion speeds (Fig. 6).

## Discussion

### Dual-layer EEG

Using dual-layer EEG we were able to improve the ability of independent component analysis to separate motion artifacts from EEG during motion. Our approach relied on additional sensor data provided by EEG electrodes that exclusively captured electrical noise and motion artifacts. Researchers have previously used signals from accelerometers to identify and remove motion artifact components from mobile EEG^68,69^, and dual-layer EEG recordings have been used to cancel electrical and motion artifacts from simultaneous EEG-fMRI recordings^57,58^ and mobile EEG^56^. By validating our combined hardware and signal processing approach with our electrical head phantom and motion platform, we demonstrated that dual-layer EEG both attenuated motion artifacts and improved the separation of ground truth input signals during motion (Fig. 3). Because we ultimately interpret spectral content of recovered electrocortical sources using time-frequency analysis, we highlight improvements in independent component analysis source separation when compared to power spectra for typical single-layer EEG recordings (Fig. 3, right column). Although independent components from single-layer EEG were relatively free of motion artifacts, greater low frequency spectral power suggests single-layer EEG independent component analysis was less effective at removing these artifacts, which ultimately degraded independent component analysis source separation (Fig. 3).

At the channel level (Fig. 3, top), single-layer EEG showed greater motion artifact susceptibility than the dual-layer EEG. In the frequency domain, motion artifacts caused greater low frequency power and an additional harmonic below the 7 Hz input signal that was not present in the dual-layer EEG channel (Fig. 3, top right). From this perspective, dual-layer EEG benefited from a passive mechanical effect of bundling wires and securing electrodes with the overlaid conductive secondary cap^56^. Nathan and Contreras-Vidal (2015) previously showed that cable bundling and an overlaid secondary cap can reduce EEG motion artifacts at slow gait speeds, but our aim was to develop solutions for studying human brain dynamics across a wide range of locomotion speeds, including running. Based on our isolated noise and electromyographic recordings, we were able to both document and remove these artifact sources. Supplementary Figures 1 and 2 show limited inter-electrode variability among individual noise and electromyography sensors, unlike single layer EEG artifacts documented by Kline and colleagues^36^. Cable bundling therefore limited inter-electrode artifact variability, and dual layer EEG enabled artifact removal.

**Figure 7:**
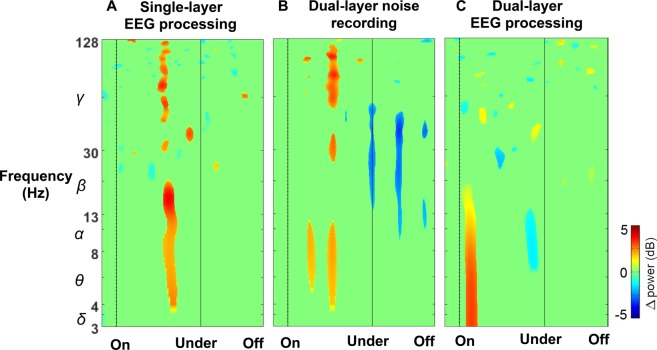
Exemplar event-related spectral perturbation plots from comparable premotor cortex clusters while subjects navigated obstacles during 1.0 m/s walking. (**A**) EEG processed without using dual-layer noise data, (**B**) Motion artifact recording from a dual-layer noise sensor, (**C**) EEG processed with dual-layer noise data.

To demonstrate that our dual-layer approach was not limited to a passive mechanical effect from improved cable bundling, we performed adaptive mixture independent component analysis on the scalp interfacing EEG electrodes alone, without relying on the noise and electromyography sensors for independent component rejection. In this supplementary analysis, we obtained three comparable cortical clusters in supplementary motor area, premotor cortex, and posterior parietal cortex after retaining independent components with equivalent current dipoles explaining greater than 85% of the scalp map. See Supplementary Figures 3 and 4 for a complete presentation of these results. Here, we show exemplar event-related spectral perturbation plots from the supplementary analysis without using the noise sensor data during adaptive mixture independent component analysis (Fig. 7A), isolated dual-layer noise recordings aggregated among subjects (Fig. 7B), and comparable dual-layer adaptive mixture independent component analysis results (Fig. 7C). Figure 7 shows incomplete motion artifact separation without using the dual layer noise sensor data, in which case it would be difficult to ascertain whether oscillations derived from true brain, or from motion-noise sources. These results provide strong support that the dual-layer approach was able to record and isolate electrocortical sources during our experiment with high fidelity, lending confidence to our assertion that the derived ensemble activity originate from true neurophysiological sources.

### Human Obstacle Navigation

Motor cortex and posterior parietal cortex have well-documented roles in planning and executing visually guided locomotor adjustments in cats^1,5^, but non-invasive human electrocortical recordings during obstacle avoidance were previously deemed impossible because of technical limitations^70^. Data from our dual-layer mobile EEG hardware and signal processing suggest supplementary motor area and premotor cortex were involved in interrupting the gait cycle nearly two steps ahead of overcoming unexpected obstacles (Fig. 6). Later posterior parietal cortex spectral power increase occurred when it would be appropriate for planning foot placement next to the obstacle (Figs 5 and 6). At faster speeds, posterior parietal cortex synchronization occurred sooner after object appearance as subjects were already in the penultimate step nearest the obstacle when the object appeared on the treadmill belt (Fig. 6).

Because visual information guides locomotor planning when navigating complex environments, sensorimotor transformations are necessary. Gibson^71^ attributed distance estimates between external objects and the body during locomotion to optic flow, and Lee^72^ further posited that the brain approximates time to contact. Neural signatures of optic flow have been linked to multiple cortical structures in mammals, including middle and medial superior temporal cortices^73,74^, premotor and motor cortices^75–77^, as well as posterior parietal cortex^78–80^. Marigold and Drew^5^ crucially identified increased neuronal discharge rates from invasive posterior parietal cortex recordings in cats while visually tracking obstacles on a treadmill belt. The authors revealed separate neuronal populations that monitored time and distance to contact, initiating their firing within a fixed range of the obstacle, independent of its speed^5^. Here, we observed later posterior parietal cortex synchronization at slower gait speeds, which maintained similar distance to contact (~0.80 m), while still providing time to plan and execute gait modifications (Fig. 5). Premotor and supplementary motor area also had synchronization at a similar distance to object contact (~1 m) (Fig. 5), but this may have been a limitation of the treadmill belt length.

For humans to identify and overcome unexpected obstacles during locomotion, a distributed cortical network is likely involved. Non-human primates have shown distance responsiveness to external stimuli in posterior parietal cortex^81,82^ and premotor cortex^83^, which has been related to avoidance behaviors^82^. Human subjects have also demonstrated prefrontal, premotor, and supplementary motor area theta event-related synchronizations while visually pursuing and intercepting objects during upper limb catching^84^, and increased cortical involvement has been shown in premotor and parietal cortices during gait adaptation^52^ and while walking with visual feedback^41^. Our subjects had limited time and space to identify and overcome unexpected obstacles, suggesting that early and prominent premotor and supplementary motor area activations were likely responsible for planning gait cycle adjustments prior to setting the support limb. Later posterior parietal cortex synchronization more likely played a role in planning limb trajectories and foot placement next to the obstacle, nearly two steps ahead of reaching the object (Fig. 6). Altered electrocortical sequencing at faster walking speeds (Fig. 5), however, might be related to interactions between visual input and the preferred human gait transition speed^85^, requiring adaptive neural strategies in an already challenging task. Further, posterior parietal cortex synchronization immediately prior to the ultimate step, rather than during the penultimate step, at 2.5 m/s running was likely due to the object’s speed, which did not allow additional steps to take place before the object was underfoot (Fig. 6). Nevertheless, consistent spectral power increases in delta, theta, and alpha bands (Fig. 4) align with frequency content of evoked potentials from EEG recordings in response to visual stimuli^86^ and balance perturbations^87^. Post-stimulus spectral power increases have been attributed to phase-resetting of ongoing electrocortical processes^86,88^, but might represent phase-locked evoked potentials here.

Since obstacles appeared randomly throughout the gait cycle in our experiment, left premotor cortex synchronization does not appear related to lower limb choice for stepping over the obstacle. However, left premotor cortex synchronization may relate to right limb dominance in our subjects. Predominant left sensorimotor theta synchronization has been observed during balance loss using mobile EEG, which the authors attributed to left hemispheric dominance during complex movements^89^ based on previous bimanual coordination studies^90^. Within the recordings from cats during locomotion, lateralization was not observed in posterior parietal cortex^5^. In contrast, posterior parietal cortex limb specificity has been observed in monkeys during reaching^91^. Marigold and Drew^5^ suggested this discrepancy in posterior parietal cortex activity was largely due to task differences between reaching and locomotion.

### Limitations and future directions

Our results differ from previous studies in a few important ways. In our data, premotor cortex and supplementary motor area synchronization typically preceded posterior parietal cortex synchronization. Intracortical recordings from cats overstepping obstacles^1^ revealed posterior parietal cortex activity occurring prior to premotor and motor cortex activity. This discrepancy may have resulted from our subjects having limited time and space to visually track approaching obstacles on the treadmill belt^92^, unlike the experiments involving cats^1,5^. Future work should examine humans walking and running overground when they can view obstacles well in advance of reaching them.

Our EEG cortical activity did not include occipital sources that would normally be involved in visual processing. It might have been expected that primary visual cortex synchronizations would have preceded right prefrontal, premotor cortex, and primary motor cortices at slow speeds, and posterior parietal cortex at fast speeds^6^. When considering the lack of primary visual cortex sources in our data, we must acknowledge the remaining challenges associated with removing muscle artifacts from scalp EEG. After applying rejection criteria to independent components based on noise and electromyography sensor spectral characteristics, we rejected occipital sources due to muscle artifact contamination. This was confirmed by our supplementary analysis performed exclusively on scalp EEG electrodes (see Supplementary Figure 4 for complete results). In this case, muscle artifacts corrupted occipital sources due to their close proximity to head stabilizing neck muscles involved in the dynamic task. Signal processing efforts to remove muscle artifacts may assist in cleaning these sources, with promising advances in the use of canonical correlation analysis and empirical mode decomposition^93,94^. Here, our neck electromyography electrodes did not include matched noise pairs in this study because we were limited to 40 noise channels in the 176-channel array (128 EEG, 8 electromyography, 40 noise). This may have limited our ability to separate motion artifacts from the electromyography data. Future assessments could include dual-layer electromyography electrodes on the neck to improve source separation with independent component analysis. Alternative channel-level cleaning approaches^56^ could also be validated using a modified electrical head phantom with neck muscle sources providing biologically realistic muscle artifacts^38,60^.

Another consideration for the recovered electocortical sources is the manner in which they were defined by our analysis. To accommodate data quantity requirements of independent component analysis, we concatenated EEG recordings from each locomotion speed, which returned spatially fixed, time varying electrocortical source signals. Potocanac and Duysens^6^ recently proposed contrasting slow and fast locomotor adjustment pathways that rely more heavily on subcortical paths during rapid online motor adjustments. Although our recovered sources showed similar involvement in supplementary motor area, premotor cortex, and posterior parietal cortex at fast locomotion speeds, it is possible that distinct cortical sources might exist in slow versus fast walking or running. In the current mobile EEG data processing pipeline, separate independent component analyses would need to be performed on slow and fast conditions, requiring longer duration recordings in each condition. Alternatively, adaptive mixture independent component analysis could be used to test for different independent component models^95^ related to slow versus fast locomotion speeds. Combining all the data into one analysis, we found prominent cortical involvement from supplementary motor area, premotor cortex, and posterior parietal cortex even at fast locomotion speeds.

## Conclusion

By using combined hardware and signal processing solutions for motion artifact removal, we found it is possible to identify human brain activity while humans stepped over obstacles while walking and running. Premotor cortex and supplementary motor area were the first cortical areas recruited to navigate unexpected obstacles. Posterior parietal cortex activity timing changed with locomotion speed, but maintained similar distance to contact when encountering obstacles, occurring nearly two steps before crossing the obstacle.

These data revealed that dual-layer EEG has the ability to enable the study of brain dynamics in a wide range of mobile real-world tasks. According to PubMed, there were over 2,800 studies on human EEG published in 2017, yet less than 1% were on mobile subjects. Motion artifacts have traditionally drastically limited the conditions in which scientists have studied human brain dynamics. As demonstrated here, adopting a dual-layer electrode approach with independent component analysis signal processing should allow new studies on human brain dynamics during physically active tasks such as real world navigation and sports studies.

## Supplementary information

Supplementary figures
