# Supplementary material for: Human electrocortical dynamics while stepping over obstacles
Source: Sci Rep. 2019 Mar 18;9:4693. doi: 10.1038/s41598-019-41131-2 (PMC6423113; doi:10.1038/s41598-019-41131-2)
Supplement: Supplementary file 1 — Supplementary figures [file 41598_2019_41131_MOESM1_ESM.pdf]

Supplementary figures

Human electrocortical dynamics while stepping over obstacles

Andrew D. Nordin [1], W. David Hairston [2], Daniel P. Ferris [1]

[1] J. Crayton Pruitt Family Department of Biomedical Engineering

University of Florida

[2] Human Research and Engineering Directorate

U.S. Army Research Laboratory

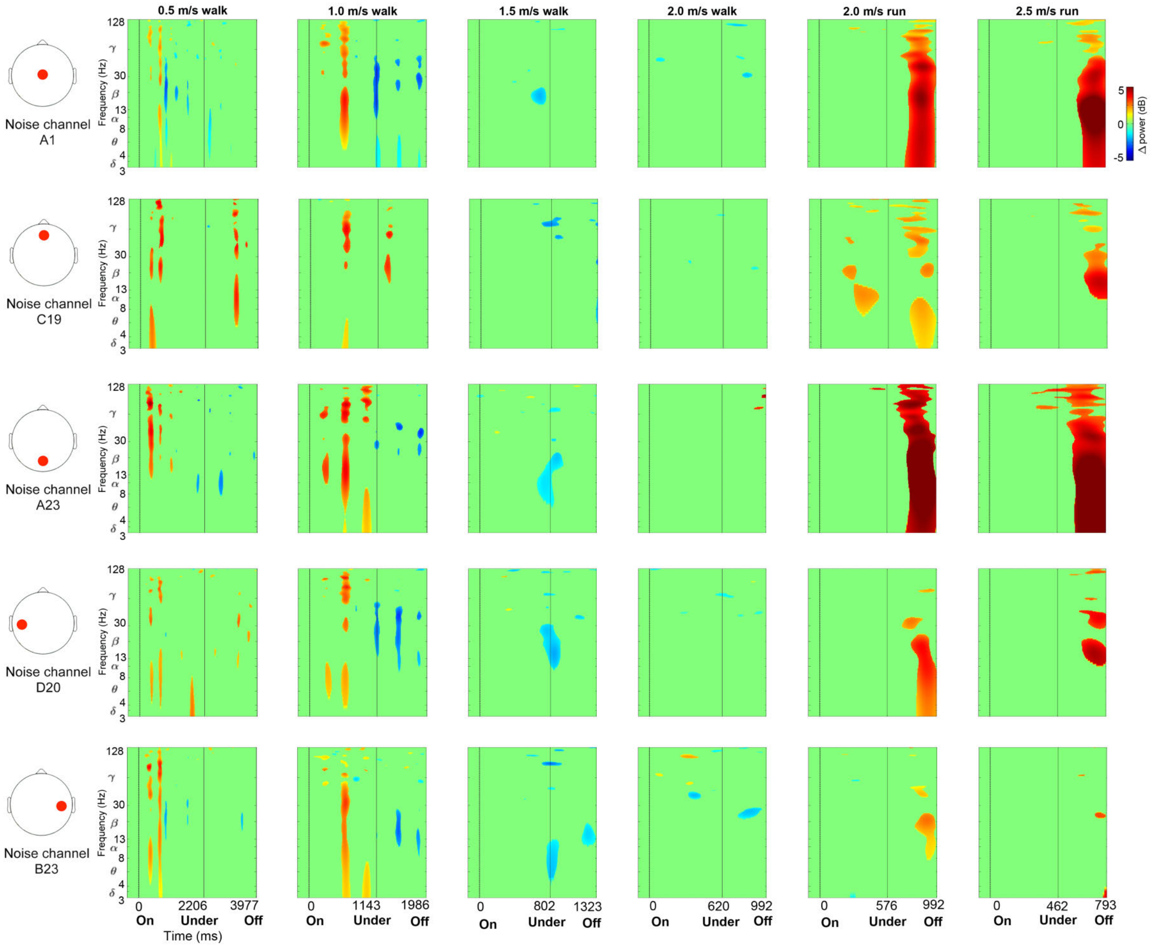

Supplementary Figure 1: Exemplar dual electrode isolated noise channel event related spectral perturbation plots at each locomotion speed (mean among subjects). Significance masked ($p < 0.05$).

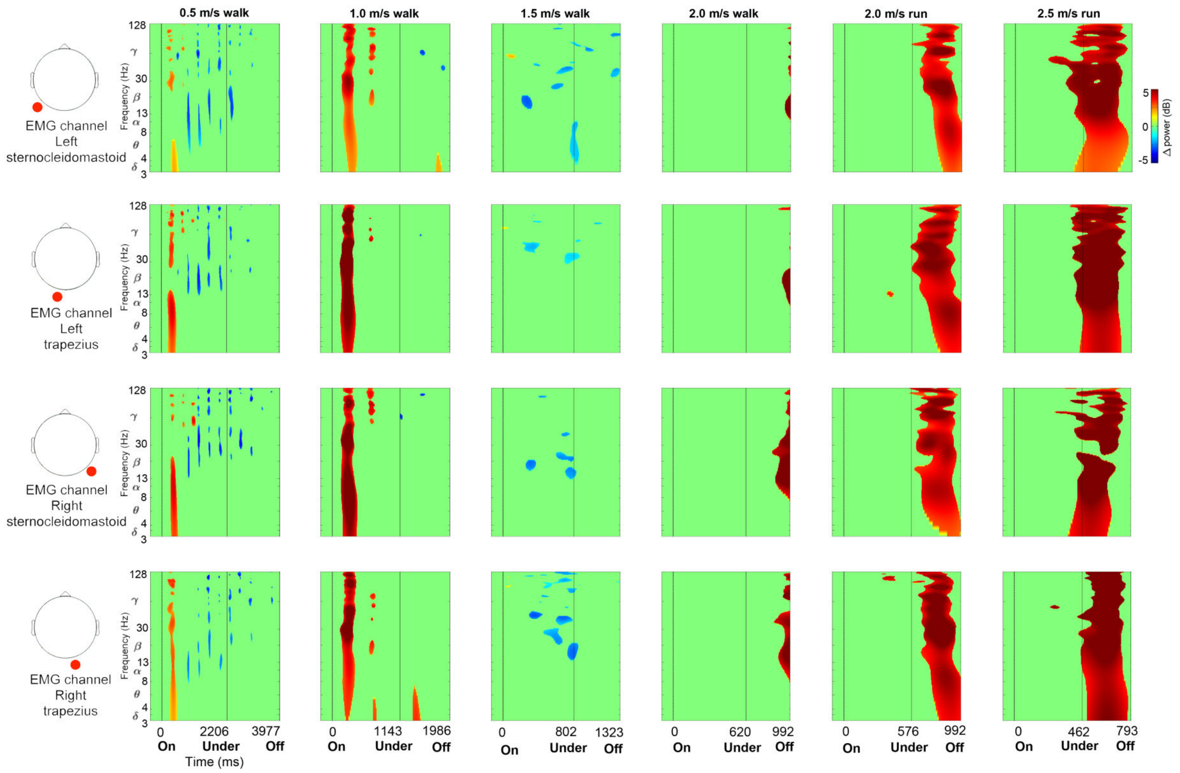

Supplementary Figure 2: Exemplar neck electromyography (EMG) channel event related spectral perturbation plots at each locomotion speed (mean among subjects). Significance masked ($p < 0.05$).

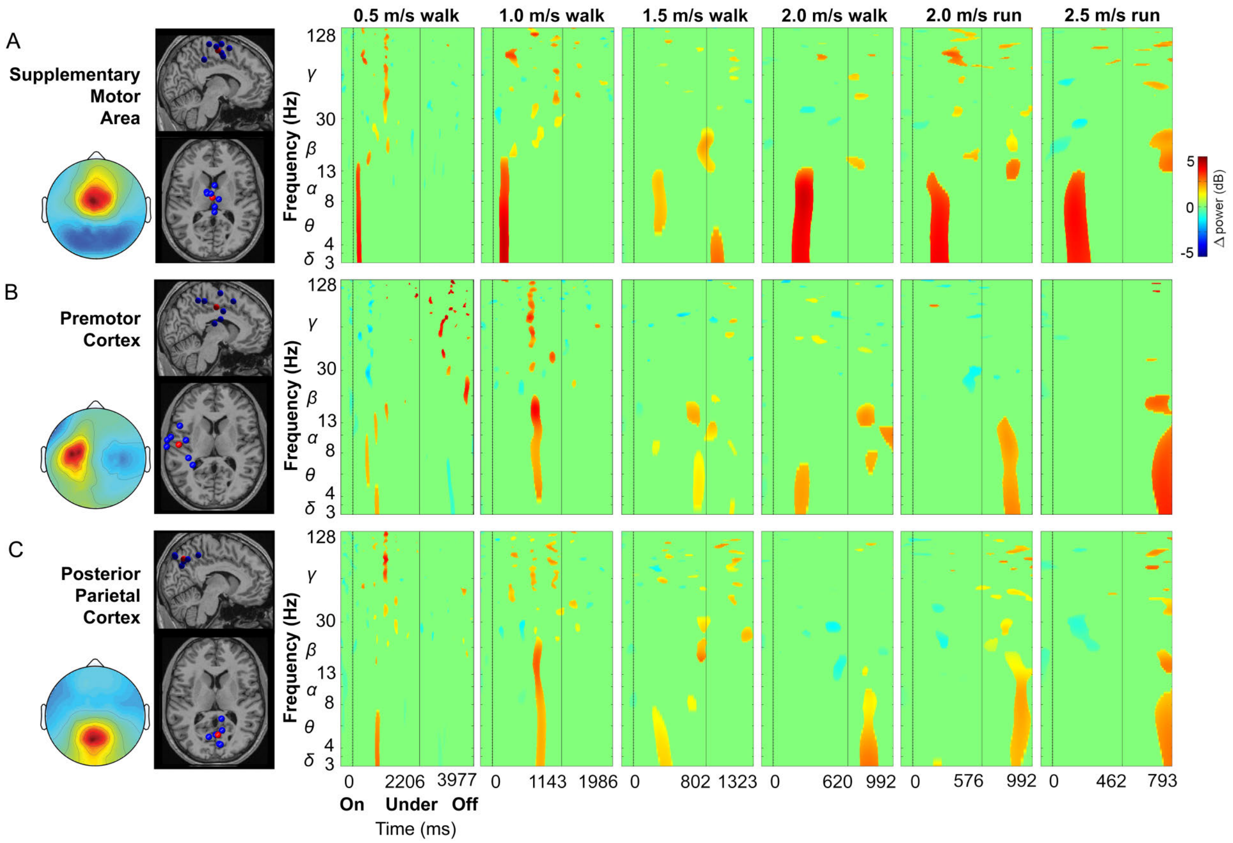

Supplementary Figure 3: Cortical clusters and event related spectral perturbation plots by speed. Data were processed without including secondary noise and electromyography sensor data in the independent component analysis decomposition and without using noise and electromyography data to reject artifact components. (**A**) Supplementary motor area (7 subjects and components), (**B**) Premotor cortex (7 subjects and components), (**C**) Posterior parietal cortex (6 subjects and components). (**Left**) Cortical cluster scalp map and dipole locations (dipole cluster centroid: red, subject components: blue) and (**Right**) mean cluster event related spectral perturbation plots at each speed. Significance masked ($p < 0.05$).

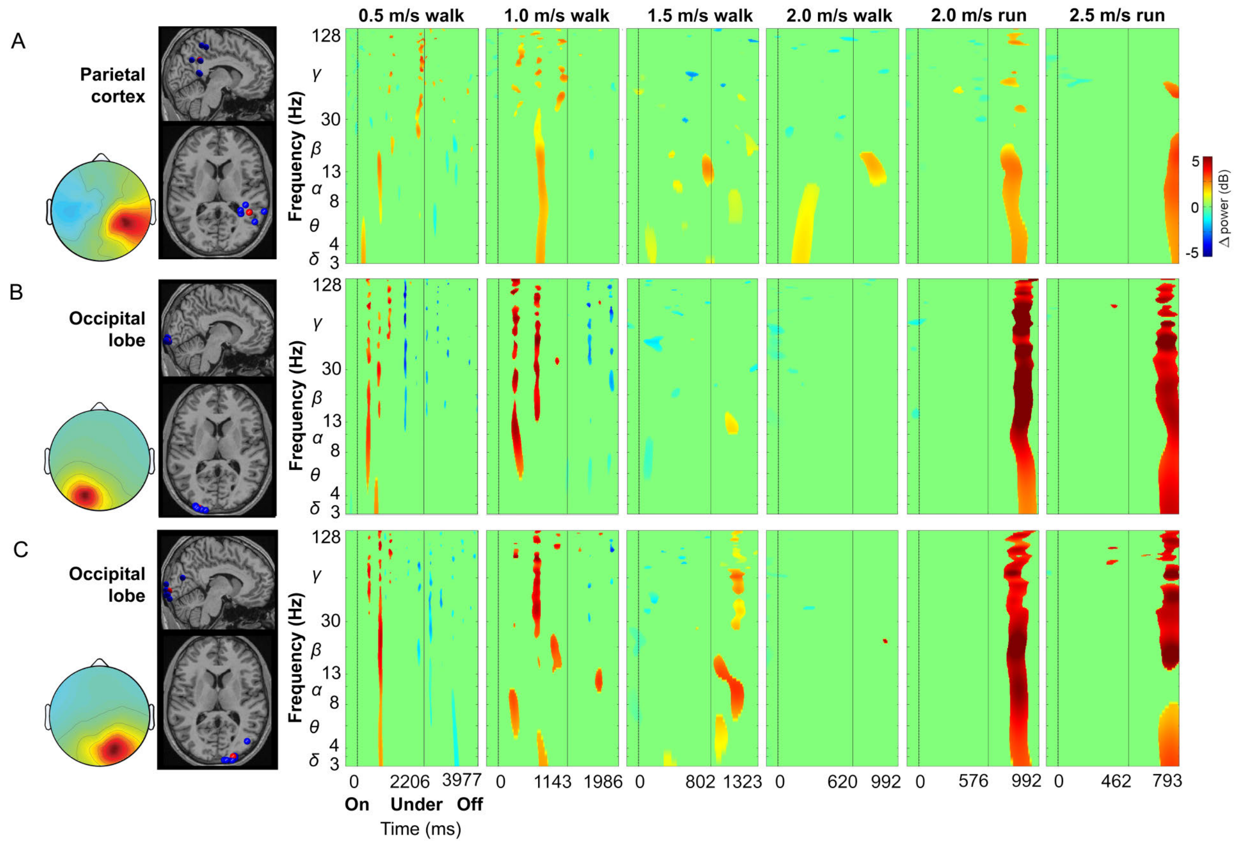

Supplementary Figure 4: Cortical clusters and event related spectral perturbation plots by speed. Data were processed without including secondary noise and electromyography sensor data in the independent component analysis decomposition and without using noise and electromyography data to reject artifact components. (**A**) Right parietal cortex (6 subjects and components), (**B**) Left occipital lobe (9 subjects and components), (**C**) Right occipital lobe (7 subjects and components). (Left) Cortical cluster scalp map and dipole locations (dipole cluster centroid: red, subject components: blue) and (Right) mean cluster event related spectral perturbation plots at each speed. Significance masked ($p < 0.05$).
